# Kernel Density Estimation (KDE) as a tool to enhance bovine tuberculosis surveillance in Santa Catarina, Brazil

**DOI:** 10.29374/2527-2179.bjvm001025

**Published:** 2025-05-09

**Authors:** Luiz Felipe Crispim Lourenço, Frederico Monfardini, Carlos Eduardo Nogueira Martins, Ricardo Evandro Mendes

**Affiliations:** 1 Programa de Pós-graduação em Produção e Sanidade Animal (PPGPSA), Instituto Federal Catarinense (IFC), Concórdia, SC, Brazil.; 2 Programa de Pós-graduação em Bioinformática, Universidade de São Paulo (USP), SP, Brazil.; 3 Instituto Federal Catarinense (IFC), Araquari, SC, Brazil.; 4 Athens Veterinary Diagnostic Laboratory (AVDL), Department of Pathology, College of Veterinary Medicine, University of Georgia, Athens, GA, United States.

**Keywords:** risk-based surveillance, cattle, spatial risk analysis, disease eradication, official veterinary service, vigilância baseada em risco, bovino, análise espacial de risco, erradicação de doença, serviço veterinário oficial

## Abstract

In areas with low bovine tuberculosis (bTB) prevalence, such as Santa Catarina state, Brazil, effective surveillance is essential for disease eradication. Current strategies may miss high-risk farms by inadequately considering spatial risk factors. This study used Kernel Density Estimation (KDE) to analyze spatial risk patterns in Santa Catarina, Brazil, leveraging the official veterinary service's (CIDASC's Sigen+ database) farm data, testing history, and animal movement records. Results revealed that while existing surveillance targets many high-risk areas, some remain unmonitored. Practices such as on-farm slaughter and insufficient movement testing create vulnerabilities that can hinder bTB detection. Integrating KDE-derived risk maps into the current surveillance efforts can improve targeted resource allocation and disease control. This study demonstrated the value of spatial risk analysis for enhancing bTB surveillance in Santa Catarina state, offering a strategic tool to support CIDASC's eradication efforts and serving as a model for other regions seeking to strengthen their surveillance programs.

## Resumo

Em áreas com baixa prevalência de tuberculose bovina (bTB), como Santa Catarina, Brasil, a vigilância eficaz é crucial para a erradicação da doença. As estratégias atuais podem não identificar fazendas de alto risco por não considerarem adequadamente os fatores de risco espaciais. Este estudo utilizou a Estimativa de Densidade de Kernel (KDE) para analisar os padrões de risco espacial em Santa Catarina, Brasil, aproveitando os dados de fazendas, histórico de testes e registros de movimentação de animais do serviço veterinário oficial (CIDASC's Sigen+ database). Os resultados revelaram que, embora a vigilância existente tenha como alvo muitas áreas de alto risco, algumas permanecem não monitoradas. Práticas como o abate na fazenda e testes de movimentação insuficientes criam vulnerabilidades que podem impedir a detecção da bTB. A integração de mapas de risco derivados de KDE aos esforços de vigilância atuais pode melhorar a alocação direcionada de recursos e o controle de doenças. Este estudo demonstrou o valor da análise de risco espacial para aprimorar a vigilância da bTB em Santa Catarina, oferecendo uma ferramenta estratégica para apoiar os esforços de erradicação da CIDASC e servindo como modelo para outras regiões que buscam fortalecer seus programas de vigilância.

## Introduction

Bovine tuberculosis (bTB) is a chronic bacterial disease that engenders substantial economic losses in livestock production, particularly within dairy cattle operations (Boland et al., 2010). As a zoonotic disease (Grout et al., 2020), bTB also presents a potential risk to human health. The primary route of transmission is respiratory, resulting in pulmonary infection and the potential manifestation of clinical signs, including coughing, lymph node enlargement, and dyspnea (Cassidy et al., 1998; Caswell & Williams, 2016; Neves et al., 2017). A significant impediment to bTB control efforts is the inherent difficulty of field diagnosis. Infected animals may exhibit only transient clinical signs or remain asymptomatic throughout their lifespan, thereby compromising detection (Downs et al., 2018).

Due to the inherent challenges in diagnosing bTB, traditional surveillance strategies have focused on on-farm testing and the inspection of carcasses for lesions at abattoirs (Kaneene et al., 2006; Siconelli et al., 2018). A common control method involves the testing and culling of infected animals. While generally effective, this approach is resource-intensive and necessitates careful planning and evaluation, especially in regions characterized by low bTB prevalence, where widespread testing may demonstrate limited cost-effectiveness (VanderWaal et al., 2017; Welby et al., 2022).

Effective disease control is predicated on the targeted surveillance of high-risk farms. Various methodologies, including prevalence studies, cattle trading network analysis, and spatial risk assessment, have been implemented to evaluate risk at the farm level. Spatial risk analysis, grounded in the principle of spatial dependency (Tobler, 1970), presents a promising approach given the expanding availability of spatially explicit data. This methodology encompasses a range of techniques—including spatial autocorrelation analysis (e.g., Moran's I), kriging, spatial scan statistics, nearest neighbor analysis, and kernel density estimation (KDE)—for the identification of high-risk areas.

Spatial risk analysis augments existing surveillance frameworks by identifying high-risk areas potentially overlooked by conventional methodologies. In 2001, Brazil launched the National Brucellosis and Tuberculosis Control and Eradication Program (PNCEBT) (Brasil, 2006), a government-led initiative. Supported by key agricultural sectors, the program implemented standardized testing, movement controls, and monitoring. Continuous studies to evaluate PNCEBT's effectiveness revealed substantial differences in bTB prevalence among Brazilian states (Bahiense et al., 2016; Dias et al., 2016; Oliveira et al., 2024; Queiroz et al., 2016; Rodrigues et al., 2022; Veloso et al., 2016). These findings directly led to the development of a document (Brasil, 2017) that classified states according to bTB risk.

Classified as a low-risk region for bTB in 2017 (Brasil, 2017), SC reported a herd prevalence of 0.5% (Veloso et al., 2016). SC's bTB control strategy, concordant with PNCEBT guidelines, incorporates mandatory bTB testing for the movement of breeding cattle, periodic testing of dairy herds, and laboratory confirmation of suspect lesions identified at abattoirs. These integrated strategies provide comprehensive monitoring of the production chain, thereby facilitating the effective identification and control of potential bTB cases.

Following the detection of a bTB outbreak on a farm, SC's surveillance protocols mandate the immediate cessation of cattle movement from the affected premises. Subsequently, the farm undergoes a test-and-cull procedure, involving the removal of all infected animals. This process is reiterated until two successive negative tests confirm the eradication of bTB from the premises (Brasil, 2006). To mitigate the economic impact of culling on farmers, SC has implemented a compensation fund, thereby promoting the program's long-term sustainability.

Further, the bTB surveillance system in SC state is committed to the eradication of the disease, moving beyond mere control. Through the implementation of rigorous surveillance protocols and comprehensive testing and control measures, the state aims to eliminate bTB, thereby safeguarding both animal and public health and contributing to national and international eradication initiatives.

Current surveillance protocols in SC are based on conventional bTB risk factors such as reproduction and dairy activity, advanced cattle age and intense cattle movements between farms (Ávila et al., 2018; Boland et al., 2010; Cardenas et al., 2021; Veloso et al., 2016) without considering spatial dependencies. This approach may result in overlooking high-risk farms located in critical areas. To mitigate this potential deficiency, we propose a methodology that cross-references high-risk areas identified through Kernel Density Estimation (KDE) with farms not currently under surveillance. This will facilitate a more targeted allocation of surveillance resources and inform future investigations into additional risk factors.

The objective of this study was to evaluate the spatial distribution of risk and its correlation with surveillance efficacy by determining whether farms identified as high-risk are proportionally represented within current surveillance efforts.

## Material and methods

Data utilized in this study were sourced from the Sigen+ database, maintained by CIDASC (Companhia de Desenvolvimento Agrícola de Santa Catarina), the official veterinary service for the state of Santa Catarina, Brazil, which includes data pertaining to bTB. Sigen+ serves as a repository for data storage, animal movement tracking, and the enforcement of livestock production regulations. Data on farm location, animal transit, bTB test results, animal age, and mortality were extracted from Sigen+ for the period spanning 2019 to 2023.

Only farms possessing complete data records throughout the study period and evidencing the presence of at least one bovine at any point during this timeframe were included in the analysis, yielding a database of 211,345 farms. Given the pioneering nature of this study within the state of Santa Catarina, an initial exploratory approach was adopted. The analysis was designed to investigate the extremes of surveillance activity, categorizing farms based on whether they were subject to any form of surveillance during the study period. Farms were designated as surveillance-subject farms (SSF) if they presented evidence of either cattle being sent to slaughter or on-farm cattle testing. Farms not meeting these criteria were classified as surveillance-ignored farms (SIF). It is pertinent to note that each officially inspected animal is considered to be under surveillance, as all suspected lesions are submitted for PCR testing. A positive result in any animal triggers mandatory testing of the entire herd.

To validate the representativeness of surveillance-subject farms (SSF) regarding the spatial risk for surveillance-ignored farms (SIF), a visual analysis of the spatial distribution of both groups within the state was conducted. Within the SSF group, control farms were defined as those with at least one animal testing positive on the comparative intradermal tuberculin test (CITT), in accordance with the PNCEBT control program guidelines.

The relative spatial risk of bTB was assessed utilizing the risk() function from the *sparr* R package. This function employs kernel density estimation on spatial data to generate a relative risk surface, thereby providing insights into the spatial distribution of bTB risk across the study area.

The kernel density estimation for the risk function can be defined as:


$$\hat{R}\left(x\right)=\frac{{\hat{f}}_{1}\left(x\right)}{{\hat{f}}_{0}\left(x\right)}$$ (1)

where:

• $\hat{R}\left(x\right)$ is the relative risk estimate at location x ;

• ${\hat{f}}_{1}\left(x\right)$ is the kernel density estimate of cases at location x ;

• ${\hat{f}}_{0}\left(x\right)$ is the kernel density estimate of controls at location x .

The kernel density estimates, ${\hat{f}}_{1}\left(x\right)$ and ${\hat{f}}_{0}\left(x\right)$ , was calculated using a fixed bandwidth h , selected using the method showed by Davies (2013), and Edge correction proposed by Diggle (2010):


$${\hat{f}}_{i}\left(x\right)=\frac{1}{n_{i}h}\overset{n_{i}}{\underset{j=1}{\sum }}K\left(\frac{x-x_{ij}}{h}\right)\cdot e\left(x,x_{ij}\right),i=0,1,$$ (2)

where:

• $n_{i}$ represents the number of observations (cases or controls);

• $K\left(\cdot \right)$ is the Gaussian kernel function;

• h is the fixed bandwidth parameter.

• $e\left(x,x_{ij}\right)$ is the edge correction.

Risk values derived from the Kernel Density Estimation (KDE) were utilized to establish classification thresholds based on quantile distribution. These thresholds were subsequently applied to assign risk categories, specifically: insignificant, low, medium, and high. The reclassified data were then converted into labeled polygons for visualization and analysis.

**Figure 1:**
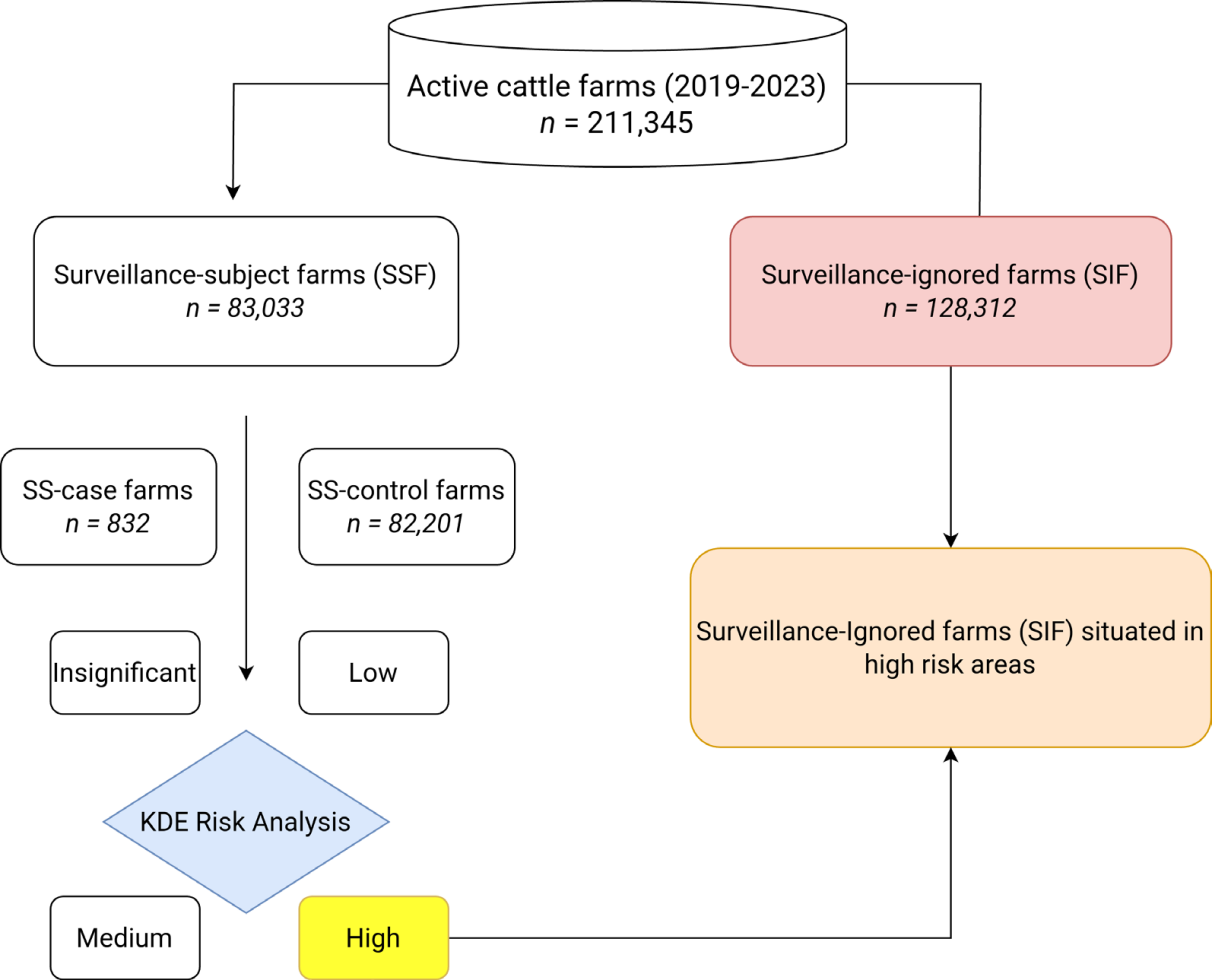
Full methodology process to obtain the results.

Following the delineation of high-risk areas based on the spatial relative risk analysis, farms located within these delineated zones but not currently under surveillance were identified. This identification process involved cross-referencing the locations of surveillance-ignored farms (SIF) with the boundaries of the high-risk areas. A comprehensive schematic of the analytical process is presented in Figure 1.

To ascertain the representativeness of surveillance-subject farm (SSF) locations relative to the overall distribution of cattle farms, a hexagonal grid composed of 49 km^2^ cells was generated, and farm location data were subsequently intersected with this grid.

## Results

**Figure 2:**
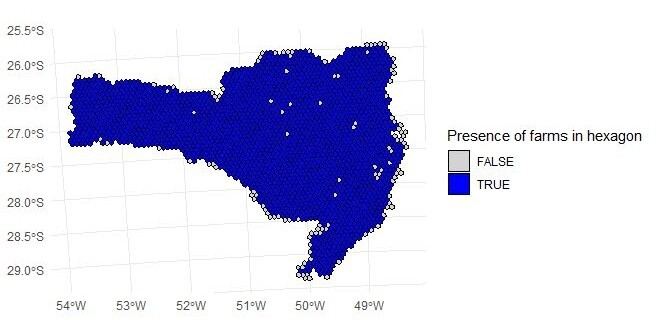
Map showing presence or absence of all cattle farms based on a grid of 49 km^2^.

**Figure 3:**
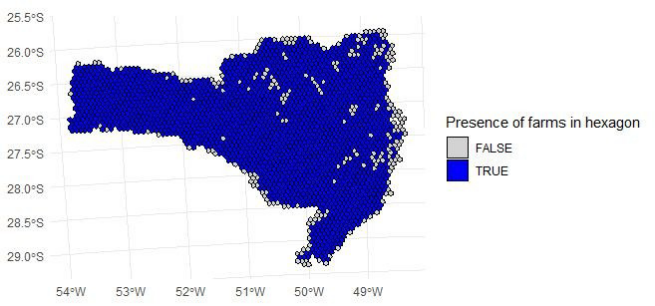
Map showing presence or absence of all SSF cattle farms based on a grid of 49 km^2^.

During the period of 2019 to 2023, a total of 83,033 farms (39.3%) underwent some form of bTB surveillance. The spatial distribution of all farms and surveillance-subject farms (SSF) is illustrated in Figures 2 and 3, respectively. Notably, the SSF farms exhibit strong spatial representativeness of the entire farm population, thus supporting the efficacy of the current surveillance coverage.

**Figure 4:**
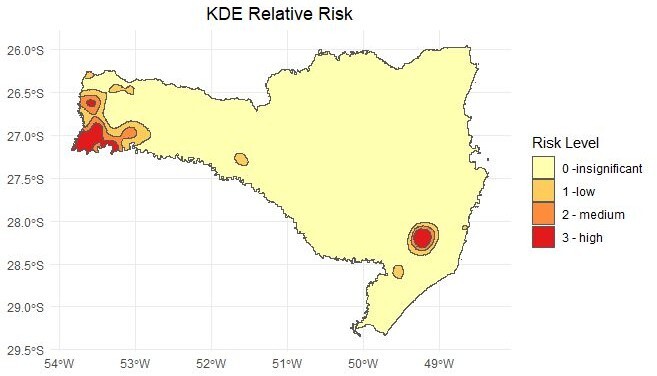
Map of risk categorization based on KDE relative risk.

The Kernel Density Estimation (KDE) analysis produced a detailed spatial risk map, presented in Figure 4. Farms were categorized into four risk levels: insignificant, low, medium, and high (Table 1). These data were then utilized to generate a cross-tabulation of farms categorized by risk level and surveillance status (Table 2).

**Table 1:** Quantiles and Risk Categories.

**Quantile Range**	**Risk Category**	**Threshold Lower**	**Threshold Upper**
-Inf - 0	Insignificant	-Inf	0
0 - 0.44	Low	0	0.44
0.44 - 0.67	Medium	0.44	0.67
0.67 - Inf	High	0.67	Inf

**Table 2:** Cross-Tabulation of Surveillance Categories and Risk Areas.

**Surveillance Categories**	**Risk Areas**			
	**0 - Insignificant**	**1 - Low**	**2 - Medium**	**3 - High**
Surveillance-ignored Farms - SIF	117447 (64%)	5077 (41%)	2385 (32%)	2045 (27%)
Surveillance-subject Farms - SSF	66372 (36%)	7407 (59%)	4962 (68%)	5650 (73%)

Results indicated a positive correlation (0.945 Pearson correlation coefficient = 0.945) between risk level and the likelihood of a farm being under surveillance. Specifically, within high-risk areas, 73% of farms were subject to surveillance protocols (Table 2), suggesting that the existing system, to some extent, incorporates risk-based criteria in its surveillance strategies.

## Discussion

The evaluation of existing surveillance systems presents considerable challenges and frequently necessitates the application of sophisticated methodologies, such as transmission models or scenario tree analysis (Guétin-Poirier et al., 2020; VanderWaal et al., 2017). The observation that 83,033 farms (39.3%) were subject to bTB surveillance between 2019 and 2023 suggests that the current system provides coverage for a substantial proportion of cattle farms within Santa Catarina.

Our findings indicate a positive correlation between risk level and the likelihood of farms being included in surveillance protocols. The fact that 73% of farms located in high-risk areas were subject to surveillance (Table 2) suggests that the system already incorporates elements of risk-based decision-making. This targeted approach enhances cost-effectiveness by concentrating surveillance efforts on farms exhibiting a higher probability of infection, thereby minimizing unnecessary testing in low-risk areas.

The risk surface generated through Kernel Density Estimation (KDE), depicted in Figure 4, revealed that high-risk areas are comparatively small in size. This observation is consistent with expectations for a region characterized by low disease prevalence, as documented in previous research (Veloso et al., 2016). This finding underscores the utility of KDE in identifying localized clusters of elevated risk that may be imperceptible through other methods. Kernel density estimation has been successfully applied in the spatial analysis of trypanosomiasis in cattle (Kivali et al., 2020), and also in estimating movements and interactions between wildlife and livestock in Africa (Rumiano et al., 2024).

However, the Kernel Density Estimation (KDE) also identified farms located within high-risk zones that were not encompassed by the current surveillance system. This discrepancy highlights a potential surveillance gap, as farms not subject to inspection may contribute to the propagation of undetected bTB cases.

By cross-referencing high-risk zones with surveillance-ignored farms (SIF), this study furnishes actionable insights for the prioritization of surveillance efforts. A significant factor contributing to these surveillance gaps is the practice of on-farm slaughter for private consumption, a practice that is not legally proscribed under Brazilian law. This practice bypasses official meat inspection protocols, potentially permitting bTB cases to remain undetected.

This strategy has been reported as efficient in several studies (Carneiro & Kaneene, 2017; García-Díez et al., 2023; Kaneene et al., 2006; Schiller et al., 2010; VanderWaal et al., 2017). This practice was observed in 38% of SIF farms. To address this issue, we recommend the implementation of educational campaigns designed to increase awareness and encourage farmers to utilize abattoirs for official meat inspection, thereby enhancing the system's capacity to detect and control bTB (McKinley et al., 2018; Neves et al., 2017).

A further challenge is presented by the inconsistent application of bTB testing protocols for cattle movement. Current regulations, as outlined in the PNCEBT guidelines (Brasil, 2006), stipulate mandatory bTB testing only for cattle intended for breeding purposes, while cattle transported for fattening are exempt from this requirement. This regulatory distinction creates a potential loophole, as farmers may misrepresent the intended purpose of cattle movements to circumvent testing expenses.

In this study, 30% of surveillance-ignored farms (SIF) were found to receive female bovines that subsequently gave birth, indicating their probable use for breeding. However, the absence of data regarding breeding bulls entering farms under the guise of fattening cattle precludes a comprehensive assessment of this potential pathway for disease transmission, suggesting that its impact may be underestimated in the present analysis. Resolution of this issue will necessitate both policy revisions and educational initiatives. Emphasis on the importance of accurate reporting, coupled with awareness campaigns highlighting the benefits of appropriate testing protocols, will contribute to the strengthening of the overall surveillance framework.

Trade in live animals among farms has been extensively documented as a significant route of bTB transmission (Berrian et al., 2012; Cardenas et al., 2021; Palisson et al., 2016), potentially exceeding the impact of local transmission (Fielding et al., 2021). However, accurately quantifying the contribution of animal trade to bTB spread is contingent upon two primary factors: the reliability of animal movement records (Ávila et al., 2018) and the sensitivity of the surveillance system (VanderWaal et al., 2017). Prompt identification of bTB outbreaks by official veterinary services is crucial for effective containment. Nevertheless, undocumented animal movements and incomplete record-keeping can substantially hinder these efforts.

Since infection probability within a herd is a function of infected and susceptible animal numbers, and the contact rate (Álvarez et al., 2014), animal movement between farms likely increases both the contact rate and the potential introduction of infected animals. Therefore, rapid bTB identification within herds, coupled with improved detection methods, is crucial for effective disease control.

## Conclusion

This study has demonstrated the efficacy of Kernel Density Estimation (KDE) as a tool for enhancing bTB surveillance by identifying spatial patterns of risk that complement conventional methodologies. Furthermore, results indicate that SC's existing surveillance system effectively targets a substantial proportion of high-risk farms. However, surveillance gaps persist, particularly among farms located in high-risk areas that are currently overlooked by control efforts. Targeted interventions, such as the promotion of official meat inspection and the implementation of pre-movement examinations, offer a promising avenue for addressing these gaps and further improving bTB control.

The findings presented here underscore the importance of integrating spatial analysis techniques, such as KDE, into established surveillance frameworks. This integrated approach facilitates more efficient resource allocation by prioritizing high-risk areas, thereby contributing to bTB eradication efforts. With these enhancements, SC can better align its bTB control strategies with national and international initiatives aimed at disease elimination, ultimately safeguarding both animal and public health.

It is anticipated that the detailed findings and insights provided by this study will inform future strategic planning for bTB surveillance programs in Santa Catarina and other Brazilian states, with the overarching objective of bTB eradication.
